# Nearly Complete Genome Sequence of Cherry Virus A, Isolated from *Prunus armeniaca* in Jammu and Kashmir, India

**DOI:** 10.1128/mra.01098-21

**Published:** 2022-03-07

**Authors:** M. Salik Noorani, Jawaid Ahmad Khan, Sheikh Khursheed, Zahid Hameed Siddiqui, Zahid Khorshid Abbas

**Affiliations:** a Department of Botany, School of Chemical and Life Sciences, Jamia Hamdard Deemed-To-Be University, New Delhi, India; b Plant Virus Laboratory, Department of Biosciences, Jamia Millia Islamia, New Delhi, India; c Ambri Apple Research Center Shopian, Faculty of Horticulture, Sher-e-Kashmir University of Agricultural Sciences and Technology, Srinagar, India; d Department of Biology, Faculty of Science, University of Tabuk, Tabuk, Saudi Arabia; KU Leuven

## Abstract

A nearly complete genome sequence of cherry virus A (CVA), isolated from the *Prunus armeniaca* plant, is presented in this publication. The genome is 7,380 bases in length and is divided into two open reading frames, with a 54-nucleotide (nt) 5′ noncoding region (NCR) and a 297-nt 3′ NCR.

## ANNOUNCEMENT

Apricot (Prunus armeniaca L.) is a widely cultivated stone fruit plant, having been domesticated in Central Asia and China before spreading to South Asia (1). In India, it is grown in Jammu and Kashmir, Himachal Pradesh, Uttaranchal, and, to a lesser extent, Punjab (2).

Cherry virus A (CVA) is a member of the *Capillovirus* genus in the *Betaflexiviridae* family. Its genome is composed of a positive-sense RNA that contains two open reading frames (ORFs). ORF1 encodes replicase and coat proteins, whereas ORF2 encodes a movement protein (MP) (3, 4). CVA has been found in all of the world's stone fruit-producing regions (5). Symptoms associated with CVA are unknown or are currently considered latent. However, CVA was isolated in 2015 from an apricot leaf sample demonstrating vein-clearing symptoms (6).

**FIG 1:**
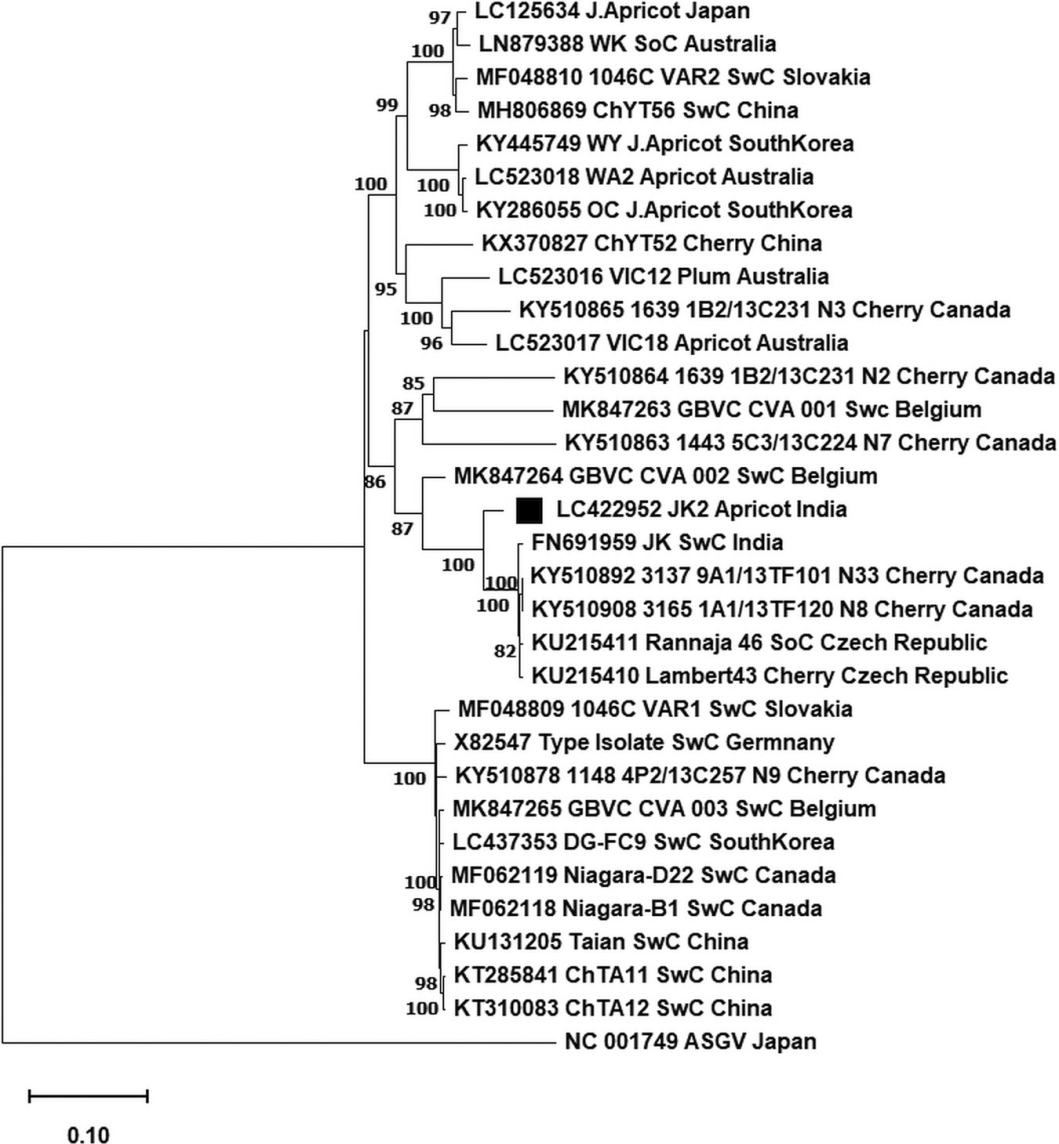
Neighbor-joining (NJ) phylogenetic analysis of 31 complete deduced nucleotide sequences of CVA with 1,000 bootstrap replicates. Bootstrap values below 70 are not shown. Each terminal node was assigned a GenBank accession number, followed by the isolate's name, host, and geographic location. SoC, sour cherry; SwC, sweet cherry; J.Apricot, Japanese apricot. Strain JK2 is marked with a black square. Apple stem-grooving virus (ASGV) was used as an outgroup. Pairwise, multiple sequence alignments and evolutionary analysis were made in MEGA 11 (10), and the resulting tree was visualized with Tree Explorer.

In this investigation, infected apricot leaves with chlorotic, short holes and necrotic symptoms were collected in May 2015 in the Srinagar district of Jammu and Kashmir. A nearly complete genome of CVA was amplified using the primer pair (CVAU and CVAL) described by Noorani et al. (7). Total RNA was extracted from 0.1 g of the infected leaves using a conventional cetyltrimethylammonium bromide (CTAB) method (8). A ProtoScript avian myeloblastosis virus (AMV) LongAmp *Taq* reverse transcription (RT)-PCR kit (New England Biolabs, USA) was used for cDNA synthesis and PCR amplification. PCR amplicons were recovered from the agarose gel using the gel extraction kit. Primer walking (Table 1) was used to sequence the isolated DNA fragments directly using the chain-terminating dideoxynucleotide Sanger sequencing method (9). Multalin (http://multalin.toulouse.inra.fr/multalin), a multiple sequence alignment tool, was used to assemble the complete genome from the individual Sanger reads. A nearly complete genome sequence of CVA was examined using the NCBI BLAST search engine (https://blast.ncbi.nlm.nih.gov/Blast.cgi) and submitted to GenBank. The strain JK2 (GenBank accession number LC422952) was 7,380 nucleotides (nt) long (A, 2,288 [31%]; T, 2,200 [31%]; G, 1,505 [20%]; C, 1,387 [18%]). The NCBI ORFfinder (https://www.ncbi.nlm.nih.gov/orffinder) predicted two ORFs from the CVA complete genome. ORF1, which encoded a 226-kDa replicase protein, spanned from nucleotides 55 to 57 (AUG start codon) to nucleotides 7078 to 7080 (ochre UAA stop codon) and translated into 2,342 amino acids. Subsequently, ORF2 (nucleotides 5400 to 6791), which encoded a 52-kDa putative MP, was predicted. The noncoding regions (NCRs) in the 5′ and 3′ regions included a total of 54 and 297 nt, respectively. A BLASTn search of the NCBI databases revealed that the JK2 strain of CVA shared 82.87% similarity with the type isolate (GenBank accession number X82547) and a maximum of 95.10% with the Canadian isolate 3137 9A1/13TF101_N33 (GenBank accession number KY510892). It also showed 94.92% similarity with the Indian sweet cherry isolate JK. Phylogenetic analysis revealed a close link between JK2 and JK isolates reported from India (Fig. 1).

**TABLE 1:** Primers used for primer walking

No.	Primer name	Primer sequence (5′ to 3′)
1	CVAU	TCACTTCCATCAATTTCCAAACAC
2	CVA558R	CAGTCAGGTTTGATGGCTC
3	CV1013F	TCAAGGGCTACAATATCAGGAC
4	CVA1920R	AAGCAAGCATTCTTCCTTG
5	CVA2751R	GTTCAAGGAGCATTCATCCA
6	CVA2638F	TTCATGACAAAATTGACTCAAG
7	CVA4596 R	TTGGCGCACATGTCATCACC
8	CVA4528F	TCCACATTCATGAAGTATGATG
9	CVA5400F	GGTTTTCCCAGTCACGAC
10	CVA6843R	CAGGAAACAGCTATGACC
11	CVAL	AAGGAAAAAGAATAAAAAGTCCTAAAGCAAGGTGC

### Data availability.

The complete genome sequence of the CVA strain JK2 has been deposited in GenBank under accession number LC422952.
